# A Life Span Perspective on Borderline Personality Disorder

**DOI:** 10.1007/s11920-019-1040-1

**Published:** 2019-06-04

**Authors:** Arjan C. Videler, Joost Hutsebaut, Julie E. M. Schulkens, Sjacko Sobczak, Sebastiaan P. J. van Alphen

**Affiliations:** 1 Clinical Centre of Excellence for Personality Disorders and Autism Spectrum Disorders in Older Adults, PersonaCura, GGz Breburg, Tilburg, The Netherlands; 2 Clinical Centre of Excellence for Body, Mind and Health, Tilburg, The Netherlands; 3 Tranzo department, Tilburg University, Tilburg, The Netherlands; 4 Viersprong Institute for Studies on Personality Disorders (VISPD), Halsteren, Bergen op Zoom, The Netherlands; 5 Clinical Center of Excellence for Personality Disorders in Older Adults, Mondriaan Hospital, Heerlen-Maastricht, Kloosterkensweg 10, PO Box 4436, 6401 CX Heerlen, The Netherlands; 6 Department of Clinical and Lifespan Psychology, Vrije Universiteit Brussel (VUB), Brussels, Belgium; 7 Department of Medical and Clinical Psychology, School of Social and Behavioural Sciences, Tilburg University, Tilburg, the Netherlands

**Keywords:** Borderline personality disorder, Life span, Course, Assessment, Treatment

## Abstract

**Purpose of Review:**

To provide an update of a life span perspective on borderline personality disorder (BPD). We address the life span course of BPD, and discuss possible implications for assessment, treatment, and research.

**Recent Findings:**

BPD first manifests itself in adolescence and can be distinguished reliably from normal adolescent development. The course of BPD from adolescence to late life is characterized by a symptomatic switch from affective dysregulation, impulsivity, and suicidality to maladaptive interpersonal functioning and enduring functional impairments, with subsequent remission and relapse. Dimensional models of BPD appear more age neutral and more useful across the entire life span. There is a need for age-specific interventions across the life span.

**Summary:**

BPD symptoms and impairments tend to wax and wane from adolescence up to old age, and presentation depends on contextual factors. Our understanding of the onset and early course of BPD is growing, but knowledge of BPD in late life is limited. Although the categorical criteria of DSM allow for reliable diagnosis of BPD in adolescence, dimensional models appear both more age neutral, and useful up to late life. To account for the fluctuating expression of BPD, and to guide development and selection of treatment across the life span, a clinical staging model for BPD holds promise.

## Introduction

The term borderline was first coined by Adolph Stern in 1938 when he identified a “border line group of patients” who “fit frankly neither into the psychotic nor into the psychoneurotic group, and are extremely difficult to handle by any psychotherapeutic method” [1]. The acceptance of borderline personality disorder (BPD) as a mental disorder in the *Diagnostic and Statistical Manual of Mental Disorders (DSM)* 3rd edition in 1980 [2] has stimulated both clinical and scientific attention. BPD is characterized by impulsivity, self-harm, suicidality, and emotional and interpersonal instability. DSM-5 [3], like DSM-IV [4], allows diagnosing BPD under the age of 18, if the symptoms are pervasive, persistent, not limited to a particular developmental stage or another mental disorder, and if the symptoms have been present for at least 1 year.

The categorical DSM concept of BPD, and of personality disorders in general, has been criticized because of its heterogeneity, diagnostic overlap with other disorders, arbitrary threshold, low reliability, and poor empirical base [5]. Factor analytic studies found support for one general factor of personality pathology underlying the nine criteria of BPD [6, 7•]. Moreover, BPD presents with many comorbid disorders [8]. Because of these limitations, a growing number of studies focuses on dimensional models of personality disorders, such as the Alternative Model of Personality Disorders (AMPD) in the DSM-5 and the new personality disorder concept of the *International Classifications of Diseases 11th Edition (ICD-11)* [9]. Both models combine a severity dimension of personality pathology and a description of five personality trait domains. In the DSM-5 AMPD, BPD is defined by negative affectivity, disinhibition and psychoticism, and several studies have indicated general support for these traits proposed for BPD [10–12]. Interestingly, ICD-11 does not retain any specific personality type with the exception of an optional specifier for “borderline pattern”, operationalized as requiring at least five out of nine criteria adapted from the DSM-5 criteria for BPD [9].

Until around 1990, therapeutic nihilism prevailed concerning the treatment options of BPD [13]. Since then, beneficial effects have been demonstrated for four comprehensive treatments: dialectical behavior therapy (DBT), mentalization-based treatment (MBT), transference-focused psychotherapy (TFP), and schema therapy [14, 15]. However, treatment studies have mainly been conducted in adults between the ages of 25 and 40, and effects remain modest and unstable at follow-up [14, 15].

Recently, a life span perspective on BPD has been introduced, stressing a lifelong vulnerability of impairments in personality functioning, including poor mentalizing and impaired social cognition, along with persisting maladaptive traits like impulsivity, emotional lability, and separation insecurity [16•]. Traits and impairments are supposed to underpin the phenomenological presentation, which may wax and wane throughout the life span, depending on the complex and changing nature-nurture interactions from early childhood onwards [16•].

This review provides an update of recent studies and viewpoints on a life span perspective on BPD, and discusses possible implications for assessment, treatment, and research. A systematic literature search was conducted for articles published between January 2014 and January 2019 using the MEDLINE and PsycINFO databases. The keyword “Borderline Personality Disorder” combined with “life span” or its synonyms (“clinical course” and “course”) yielded 145 articles. We included 33 relevant articles (clinical trials or reviews) on life span perspective, risk factors, assessment, treatment, and comorbidity of BPD. We excluded articles that did not contribute to a life span perspective or did not primarily investigate BPD, were case reports, or were written in languages other than English.

## Waxing and Waning Course of BPD from Childhood to Old Age

### Childhood and Adolescence

Until the past decade, the vast majority of our knowledge of BPD concerned diagnosis and treatment of female patients in early adulthood. Since then, BPD has also been studied more extensively in adolescents. This research points out that BPD typically first manifests itself in adolescence, and that adolescent BPD symptoms can be distinguished reliably from normative adolescent development [17]. Moreover, adolescence can be considered a particular sensitive period for BPD pathology to emerge [7•]. Two large longitudinal studies into the trajectory of BPD from childhood into young adulthood have shown that BPD pathology has its onset in the beginning of adolescence [18, 19]. Over 30% of adult BPD patients reported retrospectively that the onset of self-injurious behavior was before the age of 13, while in another 30%, this behavior started between the ages of 13 and 17 [20]. From childhood to late adolescence, vulnerable children destabilize because of a wide range of risk factors [21]. These include the following: low social economic status, stressful life events, family adversity, maternal psychopathology, cold, hostile or harsh parenting, exposure to physical or sexual abuse or neglect, low IQ, high levels of negative affectivity and impulsivity, and both internalizing (depression, anxiety, dissociation) and externalizing (attention-deficit hyperactivity disorder, oppositional defiant disorder, conduct disorder, substance use) psychopathology in childhood [21]. These risk factors predict not only BPD, but a wide range of mental disorders. Prognostic factors that are specifically associated with a BPD development in children have not yet been identified [7•, 21].

In adolescence, those individuals who do develop BPD can reliably be distinguished from those with a healthy development [22, 23]. Impulsivity, identity issues and affective instability diminish in the course of adolescence in healthy youngsters, whereas these symptoms increase over time in BPD adolescents [23–25]. The differentiation between healthy development and BPD becomes more pronounced throughout adolescence [26].

Several studies have found prevalence rates of BPD in adolescents that are similar to those in adult populations, 1–3% in community-dwelling samples, 33–49% in clinical samples and 11% in outpatient samples [27–29]. This growing empirical evidence supports that DSM-5, ICD-11, and several national treatment guidelines allow the diagnosis of BPD in adolescence [30, 31].

In sum, BPD first emerges in adolescence and symptoms mainly include impulsive behaviors and affective instability.

### Adulthood

The course of BPD from adolescence to adulthood is characterized by a symptomatic switch from predominantly symptoms of affective dysregulation, impulsivity, and suicidality to maladaptive interpersonal functioning and enduring functional impairments, with subsequent periods of remission and relapse of the full categorical BPD diagnosis, i.e., meeting the threshold of at least five out of nine DSM-criteria for BPD [16•, 32, 33]. Longitudinal studies show a general decrease of full BPD diagnoses from young to middle adulthood [34, 35]. However, remission of the categorical BPD diagnosis is commonly followed by relapse, and almost half of BPD patients never recover fully, both socially and vocationally [35, 36]. The course of core features of BPD, as assessed with retrospective questionnaires, persists throughout adulthood, such as affective symptoms (chronic dysphoria, anger, and feelings of emptiness), and interpersonal symptoms related to fears of abandonment, whereas impulsivity decreases during adulthood [35–37]. A recent cross-sectional e-diary study in everyday life showed higher affective instability prospectively between patients with BPD and healthy controls, ranging from 14 to 53 years of age, and also showed that affective instability declined with greater age in BPD [38]. Generally, the behavioral symptoms of personality disorders are less stable than the personality traits associated with BPD over time [39, 40••]. Although self-injurious and suicidal behavior decreases, risk of suicide remains as high as 10% over a 27-year course [37, 41]. Symptoms of BPD wax and wane over time, and the acute symptoms (e.g., suicidality, self-harm) change more rapidly and more readily than the temperamental symptoms (e.g., dysphoria, feelings of emptiness, and fear of abandonment) [40••].

BPD in young adulthood predicts a host of negative outcomes across the life span, including mood, anxiety, eating and substance use disorders, increased risk for physical illnesses and medical care, reduced quality of life, and reduced life expectancy [39, 42–45]. As a consequence, many BPD patients never manage to fully participate in society [34, 46].

Research on predictors of outcome of BPD, based upon the naturalistic course from adolescence into middle adulthood, has identified both positive and negative prognostic factors [40••, 46]. Predictors of good outcomes seem to be related mostly to personal capacity and competence, such as having a higher IQ, prior good full-time vocational functioning, higher levels of extraversion, higher levels of agreeableness, and lower levels of neuroticism. Predictors of poor outcomes are related to greater severity and chronicity of the disorder, higher degrees of comorbidity, and a history of childhood adversity. Non-recovered patients, which make up about 40%, experience higher rates of vocational impairment, disability, physical morbidity, and mortality than recovered patients [46].

### Late Life

Most longitudinal studies of BPD have not included people over the age of 50; because of this, our understanding of the course of BPD into late life is limited [16•]. Cross-sectional studies suggest a further decline in the prevalence of BPD from middle adulthood to old age [47, 48••]. The only ongoing longitudinal study into the prevalence and impact of personality pathology in later life, the SPAN study (St. Louis Personality and Aging Network), included patients between the ages of 55 and 64 and found a prevalence rate for BPD of 0.4%, and 0.6% if people with one criterion short for the full DSM BPD-diagnosis were included [49]. Different explanations can be pointed out for this decline in the prevalence of BPD. BPD patients, especially those that do not recover, are at elevated risk of premature death, due to suicide or other causes [50], related to an unhealthy and sometimes reckless lifestyle [51]. Furthermore, there are age differences in the expression of BPD symptoms. In a study among 1447 patients, aged 15–82 years, a significant decline was found in the externalizing aspects of BPD symptoms to the age of 50, such as impulsivity, rule breaking, and emotional turmoil, whereas abandonment fears, selfishness, lack of empathy, and manipulation remained the same [52]. In the SPAN study, three symptoms of BPD predicted interpersonal stressful life events: unstable interpersonal relationships, impulsivity, and chronic feelings of emptiness [53]. Interestingly, although impulsivity decreased with age in BPD, it continued to result in these negative consequences. BPD has also been found to predict arthritis and heart disease, in which obesity accounts for some of the variance in this relationship [44, 54].

Recent large-scale IRT analyses on data of the National Epidemiologic Survey on Alcohol and Related Conditions (NESARC) among more than 34,000 community-dwelling people, aged between 19 and 90 years, examined age differences in the likelihood of endorsing DSM-symptoms of BPD, when equating for levels of BPD symptom severity [48••]. Older people were consistently less likely to report suicidal/self-harm behavior than younger respondents and unstable/intense interpersonal relationships appeared to discriminate BPD severity better in the youngest age group compared to the oldest age group, with equivalent levels of BPD severity. It was further found that the nine DSM BPD-criteria provide substantially less information (14.7%) in older than in younger adults. Overall, these findings indicate substantial age-related differences in BPD symptom expression.

Case studies and clinical experience suggest that features of BPD can be exacerbated in old age due to contextual changes, even causing a growing prevalence of BPD in residential care and psychiatric facilities for the elderly [55–58]. Poor interpersonal functioning has caused many old BPD patients to be estranged from their family and former friends, and when they become dependent for care, this might re-trigger insecure attachment style issues and fears of abandonment [55]. BPD symptoms, together with trait neuroticism, appeared unique predictors of greater suicidal ideation in older adults, over other personality disorders and normal-range personality traits [59].

In an international Delphi study, experts in personality disorders in older adults reached consensus on the concept of “late-onset personality disorder”: a personality disorder that presents for the first time in old age as life events contribute to the expression of late-onset PD, with the major ones being death of a spouse or partner and transition to a nursing home or assisted-living facility [60]. This concept of a personality disorder emerging in late life is consistent with the ICD-11; while ICD-10 states that personality disorders tend to be stable over time, the ICD-11 guideline explicitly states that personality disorders are only “relatively” stable after young adulthood, and may change such that a person with a personality disorder in young adulthood no longer meets full criteria by middle age [9, 61]. In some cases, a person who earlier did not have a diagnosable personality disorder, may develop one later in life. Sometimes, emergence of personality disorder in older adults may be related to the loss of social supports that had previously helped to compensate for personality disturbance. Triggers for late-onset BPD could be the loss of loved-ones, which might retrigger fears of abandonment.

In sum, a life span perspective on BPD could have important implications. Instead of being a fixed set of BPD symptoms, that is invariant throughout the life span, BPD features are dynamic in nature and their expression depends on contextual and developmental factors from childhood up to old age [56]. Most BPD patients demonstrate a waxing and waning profile of impairment throughout adult life with periods of remission and relapse, while some show stable remission [40••]. This fluctuating nature of BPD should have major impact on our assessment and treatment of BPD throughout the life span.

## Assessment Implications of a Life Span Perspective

A life span perspective has two major implications for assessment of BPD. First, as the current categorical BPD diagnosis has appeared to be not age-neutral, especially in old age because of the changing expression of BPD symptoms [48••], it could be advocated to develop age-specific assessment instruments, or instruments that are age-neutral. For instance, an age-specific BPD screening instrument could be developed for the detection of BPD in older adults. The conceptualization of BPD in the AMPD in DSM-5, with levels of personality functioning (criterion A) and maladaptive trait dimensions (criterion B), has been studied for its age-neutrality in community-dwelling older adults, aged 61 and over [62]. The Short Form of the Severity Indices of Personality Problems (SIPP-SF), a questionnaire which can be used to assess criterion A, was found to be relatively age-neutral, as only 6% of the items performed differently for younger and older adults [62]. Of the Personality Inventory for DSM-5 (PID-5), which is designed to assess criterion B, only 16% of the 25 PID-5 facet-level scales showed potential age bias [63]. The brief version of this instrument, the PID-5-BF, appeared to show more age bias, as 25% of the five trait dimensions functioned differently in older adults [62]. Overall, these findings indicate that the AMPD functions similarly in older and younger adults, and is to be preferred over the current categorical model. Especially criterion A seems to be more age-neutral than criterion B.

The second implication of a life span perspective on BPD would be to develop a model that accounts for the development and possibly chronic course of BPD across the life span. Therefore, some authors have suggested a clinical staging model for BPD [56, 64••]. Staging models of diseases originated in oncology and have been developed for mental disorders, for instance for psychosis [65]. The first clinical staging model for BPD was proposed for guiding early intervention in adolescence with BPD and comorbid mood disorders [64••], and was recently elaborated to assess the severity of BPD impairment throughout the life span [56]. Clinical staging offers a description of the progression of a disorder along a continuum of disorder progression, in which progression is typically specified into five stages, from a pre-morbid stage to an end or chronic stage [66]. Clinical staging is useful for personalized selection of appropriate interventions that match with the stage of disease an individual is in. Although a typical staging profile of BPD starts in a premorbid stage in childhood and develops into a subclinical stage in early adolescence and to a first episode of full BPD in middle or late adolescence, followed by remission and relapse from middle to late adulthood, other trajectories are possible. For example, in the case of late onset BPD, people might live for many decades in a subclinical stage, and only develop significant problems, and meet full BPD criteria later in life. Another stage trajectory might be that BPD wanes into partial remission in middle adulthood, because of a relationship with a stable spouse, but re-emerges in old age, due to the destabilizing effects of bereavement, or physical decline and admittance to a nursing home. Clinical staging might shift attention towards the degree in which borderline impairment has progressed and its impact upon age-specific developmental tasks across the life span [56]. Adopting a clinical staging model across the life span could be helpful to design interventions tailored to the stage of BPD.

## Treatment Implications of a Life Span Perspective

As said, most of our knowledge of psychotherapeutic treatment of BPD comes from studies conducted in adults between the ages of 25 and 40 years, and these treatment models are focused on the acute episodes of the disorder. Typically, specialized treatments are offered rather late in the course of BPD, tend to be costly and lengthy, and available only to a subgroup of BPD patients who do seek help and manage to attend to the treatment setting [67]. Furthermore, as most existing treatments for BPD focus largely on the acute symptoms of self-harm and impulsivity, it might be fruitful to develop interventions that target underlying impairments, such as the affective symptoms, and improve social and vocational functioning, as they have been associated with recovery [40••, 46].

A life span perspective, adopting a clinical staging model, could be especially helpful to design interventions tailored to the stage of BPD. The earliest intervention is prevention of the onset of BPD by broad prevention programs. An example is preventing the transgenerational transmission of BPD, like mentalization-based treatment for parents (MBT-P) [68]. Early treatment programs target adolescents with emerging signs of BPD, such as Helping Young People Early-Cognitive Analytic Therapy (HYPE-CAT) [69]. Specific treatments have been developed for adolescents, such as DBT for adolescents (DBT-A) [70], and MBT for adolescents (MBT-A) [71]. Early intervention programs might also be developed for people with subthreshold BPD in late adulthood to prevent emerging late onset BPD, and for older adults with a first episode of acute BPD. Such treatment programs could focus on helping the older patient to adapt to age-specific stressors, like the death of a spouse or coping with becoming dependent for care. Adaptations of standard treatment programs, like MBT, DBT, TFP, and schema therapy, are needed for BPD in late life, and the first trial of schema therapy for BPD in older adults is currently being conducted [72]. Finally, specific treatment programs are needed for the frail and “old-old” BPD patients, which could be focused on staff understanding and behavioral management in care settings.

Research into the efficacy and tolerability of symptom-based pharmacotherapy for BPD [73, 74] consists of relatively few trials, and is based on findings in adults up to 50 years of age, and the quality of these studies is generally low [74]. There is a lack of research on pharmacotherapy for BPD in adolescence and in older adults. Especially in older adults, polypharmacy and changing pharmacodynamics and pharmacokinetics are complicating factors in pharmacotherapy in BPD, which can lead to side effects and interactions [75].

## Implications for Research

A life span perspective on BPD also helps defining new research objectives. One such goal would be to stop examining distal risk factors that are indicative for later general psychopathology and shed light on which precursors in childhood and adolescence are specific for BPD [7•], and what personal and contextual characteristics determine a ‘high-risk’ profile for chronic BPD. In doing so, we would be able to identify which children are at ultrahigh risk for the development of BPD.

Another major research implication of a life span perspective on BPD is to investigate whether the new dimensional models of DSM and ICD-11 indeed are capable of capturing the changing expression of BPD across the entire life span [16•]. Assessment of the AMPD with the SIPP-Sf and the PID-5 appears to be relatively age-neutral, except for the brief version of the PID-5. Therefore, the PID-BF should be examined in other populations, especially in clinical populations.

Furthermore, research could focus on the applicability of a life span clinical staging model for BPD, and on the added value of this model for selecting more appropriate interventions. The focus in treatment studies has been for too long on comparing specialized psychotherapies in adult BPD patients, but should turn to examining generic working mechanisms. Furthermore, there is a need to adapt specific treatment approaches throughout the life span, as they were designed for (young) adults and do not match with the needs of adolescents and older adults. Early intervention programs need to be developed and assessed for their efficacy across the entire life span. In the long run, early detection and intervention may prevent to a large extent that BPD evolves to a chronic stage in many cases, but for now we need to develop effective treatments for BPD in late life. This involves the adaptation of integrative treatments for older adults, but also behavioral management programs for old BPD patients in residential and home care.

## Conclusions

There is accumulating knowledge on the onset and course of BPD across the life span. Our understanding of the onset and early course of BPD is growing, but knowledge of BPD in late life is still very limited. BPD first manifests itself in adolescence, and can be distinguished reliably from normative adolescent development. BPD symptoms and impairments continue to wax and wane up to old age, and their expression depends on contextual and developmental factors. The course of BPD from adolescence to adulthood is characterized by a symptomatic switch from predominantly symptoms of affective instability and impulsivity to maladaptive interpersonal functioning and enduring functional impairments, with subsequent periods of remission and relapse of the full categorical BPD diagnosis. Although the categorical criteria of DSM allow for reliable diagnosis of BPD in adolescence, dimensional models appear both more age neutral, and especially more useful in later life. To guide early intervention and better treatment selection across the life span a clinical staging model for BPD holds promise.
